# Fecal Biomarkers of Intestinal Health and Disease in Children

**DOI:** 10.3389/fped.2014.00006

**Published:** 2014-01-28

**Authors:** Tamara Pang, Steven T. Leach, Tamarah Katz, Andrew S. Day, Chee Y. Ooi

**Affiliations:** ^1^Faculty of Medicine, School of Women’s and Children’s Health, University of New South Wales, Sydney, NSW, Australia; ^2^Department of Gastroenterology, Sydney Children’s Hospital Randwick, Sydney, NSW, Australia; ^3^Department of Nutrition and Dietetics, Sydney Children’s Hospital, Sydney, NSW, Australia; ^4^Department of Paediatrics, University of Otago, Christchurch, New Zealand

**Keywords:** inflammation, defensins, cathelicidins, lactoferrin, osteoprotegerin, calprotectin, S100A12, M2-pyruvate kinase

## Abstract

The identification of various fecal biomarkers has provided insight into the intestinal milieu. Most of these markers are associated with the innate immune system of the gut, apart from the more novel M2-pyruvate kinase. The innate immunity of the gut plays a role in maintaining a fine balance between tolerance to commensal bacteria and immune response to potential pathogens. It is a complex system, which comprises of multiple elements, including antimicrobial peptides (e.g., defensins, cathelicidins, lactoferrin, and osteoprotegerin), inflammatory proteins (e.g., calprotectin and S100A12), and microbial products (e.g., short-chain fatty acids). Dysfunction of any component can lead to the development of intestinal disease, and different diseases have been associated with different fecal levels of these biomarkers. Each fecal biomarker provides information on specific biological and disease processes. Therefore, stool quantification of these biomarkers provides a non-invasive method to define potential pathways behind the pathogenesis of diseases and can assist in the assessment and diagnosis of various gastrointestinal conditions. The abovementioned fecal biomarkers and their role in intestinal health and disease will be reviewed in this paper with a pediatric focus.

## Introduction

The innate immune system of the gut comprises of multiple elements (Table 1), each of which contributes to the fine balance between tolerance to commensal bacteria and response to potential pathogens (1). The gastrointestinal epithelium in particular, is constantly exposed to a large amount of intestinal microflora yet is able to maintain a physical barrier to exogenous stimuli while allowing the selective entry of essential nutrients (2). Its mucosal surface is covered by a mucus layer, which contains various antimicrobial peptides (AMPs) such as osteoprotegerin (OPG), defensins, and cathelicidins as well as commensal microbiota, together forming the first line of defense against pathogens. Should this mucosal barrier be breached, circulating immune cells like neutrophils and macrophages provide a second source of protection via inflammatory proteins such as lactoferrin and S100 proteins (2).

**Table 1:** **Components of the intestinal innate immune system**.

Mechanical barriers	Mucous layer (2)
	Intestinal epithelial cell layer (4)
	Intestinal motility (4)
Antimicrobial peptides	Defensins
	Cathelicidins
	Osteoprotegerin
	Lactoferrin
	Lysozyme (1)
	Secretory phospholipase A2 (1)
	Angiogenins (4)
Inflammatory proteins	Calprotectin (S100A8/S100A9)
	S100A12
Microbes and microbial products	Intestinal microflora (4)
	Short-chain fatty acids
Others	Gastric acid (3)
	Biliary and pancreatic secretions (4)
	Immune cells (neutrophils, monocyte/macrophage lineage) (2)
	Secretory IgA (4)

One mechanism through which the intestinal microbiota plays a crucial role in intestinal innate immune defense is via the production of short-chain fatty acids when colonic bacteria ferment carbohydrates (3).

**Figure 1:**
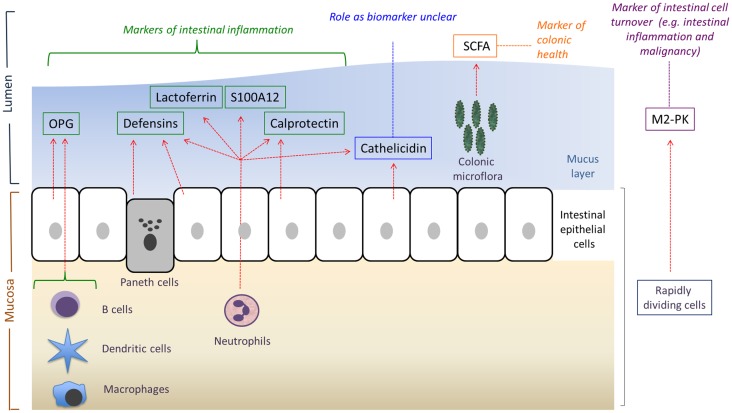
**An overview of the main sources and potential uses of the various fecal biomarkers reviewed in this article**. OPG, osteoprotegerin; SCFA, short-chain fatty acids; M2-PK, M2-pyruvate kinase. Adapted from Ref. (12).

Therefore, dysfunction of any of these components of the innate immune system can lead to impairment of the host’s mucosal defenses (4), alterations in intestinal microbial composition, and increase in the frequency and severity of intestinal infections. It has been widely hypothesized that this resultant dysbiosis can lead to gradual bacterial invasion, inflammation, and a loss of tolerance to gut bacteria (5) (Figure 1). There is abundant evidence that dysbiosis may have multiple effects on the physiology and immunology of the host, and has been associated with the development of a variety of diseases including atopy (6), obesity (7), types I and II diabetes (8, 9), cardiovascular disease (10), and inflammatory disorders (11, 12).

The identification of various intestinal AMPs, inflammatory proteins, and bacterial products has provided investigators potential insight into the intestinal milieu using non-invasive methods such as stool quantification. Understanding the role of these biomarkers in healthy (Table 2) and disease states may help characterize pathways behind disease pathogenesis, and in turn guide the development of prospective therapies.

**Table 2:** **The expression and function of defensins, cathelicidins, lactoferrin, OPG, S100 proteins, M2-pyruvate kinase (M2-PK), and short-chain fatty acids (SCFA)**.

Fecal marker	Main source and expression	Function in intestinal health
**Defensins**
α-Defensins
HNP1, 2, 3, 4	Primary granules of neutrophils (5)	
HD5, 6	Paneth cells located at base of the crypts of Lieberkühn in small intestine (*constitutive expression*) (15)	Antimicrobial peptides with microbiocidal activity (13) and chemotactic activity for immune cells (4, 17)
β-Defensins
HBD1	Colonic epithelial cells (*constitutive*) (4)	
HBD2, 3, 4	Colonic epithelial cells (*inducible*) (15)	

**Cathelicidins**	Neutrophils, keratinocytes, epithelial cells of respiratory, urogenital, and gastrointestinal tract (37)	
	Especially lower small intestine and colon (15) Expression in colon is *constitutive*. Remains unclear if expression is determined by differentiation of colonocytes (30, 37)	Antimicrobial peptides with microbiocidal activity (13) and chemotactic activity for immune cells (4, 17)

**Lactoferrin**	Mucosal epithelial cells and secondary granules of neutrophils (45)	
	*Constitutive* expression by mucosal epithelial cells (45) *Inducible* during inflammation (45)	Multiple roles including antimicrobial (42) and immunomodulatory activity (45)

**Osteoprotegerin**	Intestinal epithelial cells, osteoblasts, dendritic cells, macrophages, B-lymphocytes, bone marrow stromal cells (52)	Anti-inflammatory effects when bound to RANKL (51)
	*Constitutive* expression by colonic epithelial cells (51)	Pro-inflammatory effects when bound to TRAIL (54)
	*Inducible* during inflammation (51)	

**S100 proteins**
S100A8/S100A9 (calprotectin)	Cytoplasm of neutrophils, monocytes and epithelial cells (*inducible*) (58)	Pro-inflammatory role in innate immunity by acting as DAMPs (60)
S100A12	Cytoplasm of neutrophils (*inducible*) (65)	

**M2-PK**	Expressed by all rapidly dividing cells (83)	Key enzyme in the glycolytic pathway (83)

**SCFA**	Produced upon fermentation of complex carbohydrates by anaerobic microflora in the colon (91)	Multiple roles including: energy source for colonocytes (91). Regulation of fluid and electrolyte uptake (91). Colonic microbiota homeostasis (3)

*HNP, human neutrophil peptide; HD, human defensin; HBD, human β-defensin; RANKL, receptor activator of NF-κB ligand; TRAIL, tumor necrosis factor (TNF)-related apoptosis-inducing ligand; DAMPs, damage-associated molecular pattern molecules*.

Many fecal biomarkers have been identified to date but only a few have been more extensively studied in children. Hence in this review, we aim to provide an overview of the roles of defensins, cathelicidins, lactoferrin, OPG, S100 proteins, SCFA, and the more novel M2-pyruvate kinase (M2-PK) in health and various disease states in the pediatric population. The practical aspects and limitations of fecal biomarkers are also discussed. We performed a search of the databases Medline, EMBASE, and PubMed for articles written in English, including review articles, related to the relevant fecal biomarkers in children, published after the year 1980.

### Defensins

Defensins are AMPs that are divided into two main subfamilies based on structure: α- and β-defensins (4). To date, 10 human defensins have been identified (5), further details of which are discussed in Table 2.

### Role in health

As AMPs, defensins exhibit microbiocidal activity by forming micropores in the membrane, causing loss of structural integrity and eventually cell lysis (13). Via this mechanism, defensins are microbiocidal against Gram-positive and Gram-negative bacteria, fungi, viruses, and protozoa (14) thereby protecting the intestinal epithelium and stem cells from pathogens as well as regulating the number and composition of commensal bacteria (5).

#### α-Defensins

The microbiocidal activity of α-defensins was demonstrated in a cohort study of African adults, which showed that low Paneth cell α-defensin-gene expression was associated with a higher risk of diarrheal infections (15). Interestingly, although human α-defensins (HD) 5 and 6 are largely confined to the small intestine, they are also secreted in the colon of patients with ulcerative colitis (UC) due to the presence of metaplastic Paneth cells. This is thought to provide an alternative “on-demand” mechanism that provides antimicrobial expression and protection of the gut (14).

Apart from their microbiocidal role in the innate immune system, α-defensins are chemotactic for monocytes, dendritic, and T cells, thereby providing regulation of adaptive immunity via activation and recruitment of adaptive immune cells (4). Furthermore, α-defensins 1–4, known also as human neutrophil peptides (HNPs), enhance the expression of TNF-α and IL-1β in activated human monocytes and reduce the expression of vascular cell adhesion molecule (VCAM)-1 in human endothelial cells activated by TNF-α. This regulation of cytokine production and adhesion molecule expression indicates a potential role of HNPs in modulating inflammatory responses (16).

#### β-Defensins

The antimicrobial function of β-defensins has also been well established. During health, the constitutive expression of human β-defensin (HBD) 1 by epithelial cells prevents microbial invasion by strengthening the intestinal mucosal barrier. The induction of HBD2, 3, and 4 during inflammation or infection may prevent further bacterial entry into an already compromised epithelium and contribute to antimicrobial defense during inflammation at this site (14). Like the α-defensins, β-defensins also exhibit chemotactic activity for immature dendritic cells (DCs) and memory T cells through the CC chemokine receptor type 6 (CCR6), thus promoting adaptive immune responses by recruiting these cells to the site of microbial invasion (17).

### Role in intestinal disease

#### Inflammatory bowel disease

The expression of defensins in intestinal inflammation has been extensively studied, especially in the setting of inflammatory bowel disease (IBD) and its two main subsets, UC and Crohn’s disease (CD). There is a consensus in the literature that in UC, which involves superficial inflammation confined to the colonic mucosa, increased HBD2 and Paneth cell α-defensin expressions are characteristic (18). HBD2 levels were elevated in stool collected from children with active UC (median 356 ng/g, range 40–527) compared to healthy controls (median 13 ng/g, range 3–56; *P* = 0.0002) (19).

In colonic CD, there is attenuated induction of β-defensins as measured by mucosal mRNA (20). In ileal CD however, reduced Paneth cell α-defensin expression was observed in ileal biopsies (21). Kapel and colleagues (19) found only a three- to fourfold increase in fecal HBD2 levels in children with CD as opposed to the abovementioned >10-fold increase in fecal HBD2 levels for UC. This impaired induction of β-defensins in colonic CD has been attributed to low β-defensin-gene copy number (22), while other studies have suggested an association between CARD15/NOD2 mutations and HBD2 deficiency (23). The α-defensin deficiency in ileal CD has also been linked to NOD2 mutations (5). An important finding from the study by Kapel et al. (19) was a positive correlation between fecal calprotectin and HBD2, suggesting that fecal HBD2 is associated with intestinal inflammation.

#### Irritable bowel syndrome

Irritable bowel syndrome (IBS) is a very common functional bowel disorder in the absence of macroscopic and histologic inflammation, characterized by abdominal pain and altered bowel habits (24). According to the ROME III criteria, IBS can be subtyped into diarrhea predominant (IBS-D), constipation predominant (IBS-C), mixed diarrhea and constipation (IBS-M), and unsubtyped IBS (IBS-U) (25). There is mounting evidence that microbial dysbiosis is associated with IBS, with the implication of small intestinal bacterial overgrowth (SIBO) in its pathogenesis (26). This alteration in gut microbiota is hypothesized to lead to activation of the mucosal innate immune response (26), which has been supported by various studies. In particular, a study by Langhorst and co-workers comparing fecal HBD2 in patients with IBS, active UC, and healthy controls, found that fecal HBD2 was significantly elevated in the IBS group (mean ± SD: 76.0 ± 67.9 ng/g) in comparison to controls (29.9 ± 16.1 ng/g; *P* < 0.001), although to a lesser extent than patients with active UC (106.9 ± 91.5 ng/g). Their findings support the hypothesis of an activation of the mucosal innate defense in IBS toward low-grade mucosal inflammatory activity (24), which is in turn supported by studies that have found increased mast cells (27) and colonic lamina propria immune cells in patients with IBS (28).

#### Necrotizing enterocolitis

Necrotizing enterocolitis (NEC) is one of the most common gastrointestinal emergencies in neonates (29). Its main risk factors are prematurity and low birthweight (29). The pathogenesis of NEC remains elusive but there is strong evidence that inappropriate bacterial colonization of the neonatal gut plays a role (30). This has been supported by various studies showing that probiotic supplementation in preterm neonates of very low birthweight (<1500 g) reduces the risk and mortality of NEC (31). Like IBS, this aberrant postnatal bacterial colonization may lead to an activation of the innate immune system of the gut.

A study by Jenke and colleagues aimed to assess this intestinal mucosal innate response via HBD2 expression in extremely low-birth-weight (ELBW) infants with NEC (32). They found that infants with moderate NEC had elevated fecal HBD2 concentrations before onset of symptoms, probably reflecting an adequate immune response (32). However, infants with severe NEC showed no increase in fecal HBD2 concentrations before and during the disease. This finding together with a lack of increase in fecal calprotectin concentration and normal villin expression, the latter of which is reduced with epithelial cell loss, suggests a specific deficiency of innate defense activation in ELBW infants rather than an impaired intestinal epithelial barrier, leading to a more severe course of NEC (32).

## Cathelicidins

Cathelicidins are a family of precursor proteins with a well-conserved cathelin pro-region, followed by a highly variable C-terminal antimicrobial domain (33). Human cationic antimicrobial protein 18 (hCAP18) is the only human cathelicidin precursor protein, which is cleaved from the cathelin pro-region to produce the mature cathelicidin peptide LL-37 (33). The expression of cathelicidin is summarized in Table 2.

### Role in health

Proteolytic cleavage of hCAP18 into LL-37 is required for bactericidal activity. Like defensins and other AMPs, cathelicidin exhibits microbiocidal activity by disrupting microbial membrane integrity (13). *In vitro* studies have shown activity against a range of Gram-negative and Gram-positive bacteria including gastrointestinal pathogens such as *Helicobacter*, *Salmonella*, *Shigella*, and the fungus *Candida albicans* (15). Its antibacterial activity is mediated by the lipopolysaccharide (LPS)-binding and neutralizing properties of cathelicidin, thereby inhibiting LPS-induced cellular responses, such as the release of TNF-α, nitric oxide, and tissue factor (34).

Cathelicidin contributes to host defenses by playing a role in the inflammatory process. It exhibits *in vitro* chemotactic activity for the selective migration of human peripheral blood monocytes, neutrophils, and CD4^+^ T cells (35). Cathelicidin is also chemotactic for mast cells, inducing their degranulation, resulting in the release of inflammatory mediators like neutrophil chemo-attractants and histamine, which increases vascular permeability, thus further facilitating neutrophil infiltration of inflamed tissue (36).

In addition, cathelicidin has been reported to help in the repair of damaged tissue and wound closure by promoting wound neo-vascularization and re-epithelialization of healing skin (34); its role in intestinal mucosal healing is unknown.

### Role in intestinal disease

#### Inflammatory bowel disease

A study looking at cathelicidin expression in colonic mucosal biopsies of UC, CD, and healthy patients found a significantly higher expression in patients with UC when compared to those with CD (33). However, when inflamed and non-inflamed mucosa of patients with UC or CD was compared, no difference in expression was found. In addition, increased CD4 expression levels (a surrogate marker of infiltrating immune cells) in inflamed CD mucosa were not associated with increased cathelicidin expression (33). These findings suggest the dissociation between cathelicidin expression and inflammation. Other studies have supported this by showing that pro-inflammatory mediators do not upregulate cathelicidin expression, whether *in vitro* or *in vivo* (37, 38).

Regulation of cathelicidin expression is unclear. Cathelicidin expression was reported to be regulated by butyrate (a SCFA) through butyrate-induced differentiation of colonic epithelial cells (15, 37). However, Schauber and colleagues (33) showed that butyrate-enhanced colonocyte differentiation and butyrate-induced cathelicidin expression are regulated separately via distinct signaling pathways.

#### Shigellosis

Shigellosis is a major cause of morbidity and mortality in developing countries (39). It is caused by infection with the highly contagious *Shigella* species, which invades the colonic mucosa causing inflammation that destroys the mucosal barrier (40). The clinical manifestations are the passage of bloody mucoid loose stools, abdominal cramps, rectal tenesmus, and fever (40).

Reduced cathelicidin levels have been observed in gut biopsies of patients with shigellosis (41). It has been suggested that this down-regulation is a strategy by pathogenic microbes to increase their virulence by circumventing host immune defenses. Adjunct therapy with butyrate during shigellosis resulted in enhanced expression of cathelicidin in rectal epithelia, prolonged cathelicidin secretion in stool, and early reduction in inflammation (40).

## Lactoferrin

Lactoferrin is an iron-binding glycoprotein of the transferrin family which plays a role in transporting serum iron (42). The expression pattern of lactoferrin (Table 2) indicates that it may play a role in the innate immune response (43).

### Role in health

Lactoferrin has multiple roles, some attributable to its iron-binding properties. Interestingly, lactoferrin is both promicrobial and antimicrobial, the former because of its ability to provide iron to bacteria. In contrast, its bacteriostatic activity is due to the sequestration of iron and subsequent deprivation of this nutrient from pathogenic bacteria (42). Furthermore, independent of its iron-binding properties, lactoferrin possesses bactericidal activity via direct interaction with bacteria (43). It was observed that apolactoferrin, the iron-free form of lactoferrin, can bind to the outer membrane of Gram-negative bacteria to cause the rapid release of LPS and an increase in membrane permeability (44). In addition, it is widely accepted that lactoferrin has antiviral, antifungal, and antiparasitic functions (42).

Lactoferrin is a modulator of the innate and adaptive immune system. Its anti-inflammatory activity is attributed to the inhibition of cytokines such as TNF-α and IL-1β (45). It has also been suggested that lactoferrin induces immunity via activation of various signaling pathways. Its positive charge allows it to bind to negatively charged molecules on the surface of various immune cells and this association may trigger signaling pathways that lead to cellular activation, proliferation, and differentiation. Lactoferrin is also transported into the nucleus where it can bind DNA and activate different signaling pathways (42).

### Role in intestinal disease

#### Inflammatory bowel disease

An increase in fecal lactoferrin levels occurs during intestinal inflammation (46) due to mucosal infiltration and degranulation of neutrophils, providing an additional source of lactoferrin to aid the mucosal innate response (45). Elevated fecal lactoferrin levels have been reported in IBD (47) with a sensitivity of 78%, and specificity of 90% in identifying inflammation in adults with chronic UC and CD (46). In addition, fecal lactoferrin showed good correlation to disease activity (endoscopic and histopathologic) and was 100% specific in ruling out IBS (46). Using an established cutoff point of 7.25 μg/mL for patients with IBD (48), similar findings of elevated fecal lactoferrin in pediatric patients with UC (1880 ± 565 μg/mL) and CD (1701 ± 382 μg/mL) compared to healthy controls (1.17 ± 0.47 μg/mL; *P* < 0.001) were observed (49). Fecal lactoferrin also correlated well with clinical activity indices and erythrocyte sedimentation rate (ESR) (49).

## Osteoprotegerin

Osteoprotegerin is a member of the tumor necrosis factor (TNF) receptor superfamily and functions as a soluble decoy receptor of the receptor activator of NF-κB ligand (RANKL) and TNF-related apoptosis-inducing ligand (TRAIL) (Table 2) (50, 51).

### Role in health

#### Receptor activator of NF-κB ligand

Osteoprotegerin is best known for its role in bone metabolism. It binds to RANKL and blocks its interaction with RANK, thereby inhibiting osteoclastogenesis (52). In addition, the RANK/RANKL/OPG system has a role in regulating intestinal inflammation by modulation of colonic DCs. In a murine model, exogenous OPG reduced DC survival, eliminating the antigen-presenting cell (APC) for colonic CD4^+^ T cells, thereby reducing inflammation (53).

#### TNF-related apoptosis-inducing ligand

TNF-related apoptosis-inducing ligand is a member of the TNF ligand superfamily that induces cellular apoptosis. The interaction of OPG with TRAIL during intestinal inflammation inhibits apoptosis of DCs and activated T cells, thereby perpetuating intestinal immune activation (54). This pro-inflammatory effect opposes the above findings of a potent anti-inflammatory effect of OPG when interacting with RANKL (53).

### Role in intestinal disease

#### Inflammatory bowel disease

The role of OPG in intestinal inflammation was affirmed by a study of children with newly diagnosed CD that showed elevated serum and intestinal mucosal OPG levels (52). Importantly, fecal OPG was also raised in moderate/severe CD (6463 ± 8691 pg/mL) and mild CD (477 ± 848 pg/mL) when compared to healthy controls (63 ± 0.001 pg/mL; *P* < 0.0001). It was proposed that the excess circulating OPG was a result of increased mucosal OPG production due to inflammation. In addition, serum and fecal OPG decreased after treatment with exclusive enteral nutrition (EEN). This indicates that fecal OPG can be used as a marker of mucosal OPG expression and intestinal inflammatory severity in CD.

A recent study has provided further insight into the role of OPG in intestinal inflammation. Via *in vitro* methods, Nahidi and co-workers (55) found that OPG possesses pro-inflammatory properties via its induction of gut barrier dysfunction and secretion of pro-inflammatory cytokines. Their results also provide evidence that OPG, like TNF-α, exerts its pro-inflammatory effects by NF-κB activation.

#### Cryptosporidiosis

Cryptosporidiosis is caused by infection with the waterborne protozoan parasite *Cryptosporidium* (56). It characteristically results in watery diarrhea (56) that is usually self-limited in immunocompetent individuals (57) but may be profuse and prolonged in immunocompromised patients (56).

The “disease-promoting” effect of OPG mediated by TRAIL, as discussed above, was further alluded to in an *in vitro* study of human ileal mucosal cells infected with *Cryptosporidium* (57). The results showed that treatment with TRAIL induced epithelial cell apoptosis and reduced parasite numbers. However, giving recombinant OPG blocked these therapeutic effects. Moreover, this study showed an early increase in OPG expression by the infected epithelial cells, suggesting that *Cryptosporidium* may upregulate OPG to protect against early apoptosis by TRAIL.

## S100 Proteins

S100 proteins are a family of more than 20 calcium-binding proteins (58). Unlike many of the other S100 proteins that exert their regulatory effects in a Ca^2+^-dependent manner solely within the cells they are expressed, three S100 proteins – S100A8, S100A9, and S100A12, have also been found to have extracellular activity (59). These three members are, moreover, specifically associated with innate immune functions due to their expression in phagocytes (60). S100A8 and S100A9 associate to form a complex known as calprotectin (58). The expression of calprotectin and S100A12 is summarized in Table 2.

### Role in health

Calprotectin (S100A8/S100A9) and S100A12 have a pro-inflammatory role in innate immunity and are part of a group called damage-associated molecular pattern molecules (DAMPs), due to their release by activated or damaged cells under conditions of cellular stress (60). An emerging concept of pattern recognition involves sensing of exogenous pathogen-associated molecular patterns (PAMPs) and endogenous DAMPs via the multi-ligand receptor for advanced glycation end products (RAGE) and toll-like receptors (TLRs), enabling innate immunity to achieve our primary host defense against invading microorganisms and non-specific stress factors (58, 60).

In accordance with their pro-inflammatory role, calprotectin and S100A12 are significantly overexpressed at sites of inflammation, and there is a strong correlation of their serum concentrations to inflammation (60). The secretion of calprotectin by phagocytes is induced when phagocytes come into contact with inflamed endothelium. One mechanism that calprotectin is thought to promote inflammation is via induction of pro-inflammatory chemokines, adhesion molecules (e.g., VCAM-1 and ICAM-1) and β_2_-integrin, thereby mediating leukocyte recruitment, adhesion, and transendothelial migration to inflamed tissue (58, 61).

S100A12 has also been shown to mediate inflammation via the induction of similar adhesion molecules to calprotectin and it also upregulates the production of pro-inflammatory cytokines by macrophages, including TNF-α and IL-1β (58). Moreover, it has been implicated in a novel pro-inflammatory axis binding RAGE, leading to the transduction of pro-inflammatory signals in the endothelium and immune cells (60).

The pediatric reference range for fecal calprotectin was established in a study of 117 healthy children and found a median of 13.6 μg/g (95% confidence interval, 9.9–19.5 μg/g) (62). It was also suggested that the adult cutoff level for intestinal inflammation of 50 μg/g can be applied to children as well (62). More recently, a pediatric reference range for S100A12 has also been determined in a study involving 56 healthy children (63). A median of 0.5 mg/kg (range 0.39–25 mg/kg) was found, suggesting that the established adult cutoff of 10 mg/kg can also be applied to children (63).

### Role in intestinal disease

#### Inflammatory bowel disease

S100 proteins, especially calprotectin and S100A12, have been extensively studied in both the adult and pediatric IBD populations. Serum and mucosal levels of both of these biomarkers have been shown to be elevated in children with IBD (64).

Fecal calprotectin levels are also significantly elevated in children with IBD (median 1265 mg/kg) compared to children without IBD (median 30.5 mg/kg; *P* < 0.0001). A sensitivity of 100% and specificity of 67% was found for fecal calprotectin in identifying children with IBD (cutoff >50 mg/kg for IBD) (65). In addition, multiple studies have shown that fecal calprotectin correlates closely with endoscopic and histological grading of colonic inflammation in both UC and CD (66–68). A positive correlation between fecal calprotectin and clinical activity indices in both CD and UC has also been demonstrated (69) and therefore, fecal calprotectin has been proposed as a useful tool in monitoring disease activity in children with IBD (67).

Together, there is substantial evidence in the literature that fecal calprotectin is a sensitive marker of intestinal inflammation. Multiple studies have shown that fecal calprotectin can differentiate IBD from functional disorders like IBS (69, 70), with validation in children (67, 71). Based on a cutoff of 30 mg/kg, fecal calprotectin discriminated adults with active CD from those with IBS with 100% sensitivity and 97% specificity (72). However, it is not a disease-specific fecal marker and is also elevated in other gastrointestinal disorders like gastroenteritis (73) and colorectal cancer (CRC) (70), as well as during non-steroidal and non-inflammatory drug use (74).

Another potential aspect of fecal calprotectin is in predicting relapse in children with IBD. An elevated calprotectin level in stool was found to be associated with a 13-fold increased risk of relapse in adult IBD patients experiencing remission (75), with another study suggesting that fecal calprotectin is more accurate in predicting relapse in UC than CD (76).

S100A12 is also elevated in stool from children with active IBD (median 95.40 mg/kg; range 6.19–349.9 mg/kg) compared to healthy controls (median 0.69 mg/kg; range 0.39–17.73 mg/kg; *P* < 0.0001) (77). Moreover, it was found to have a sensitivity of 96% and specificity of 92% in distinguishing children with active IBD from healthy controls when a 10 mg/kg fecal S100A12 was used as a cutoff.

#### Cystic fibrosis

Cystic fibrosis (CF) is the most common life-shortening autosomal recessive disease in Caucasians, with an incidence of 1 in 2500 live births (78). There is evidence that CF predisposes to inflammatory changes not only in the respiratory system but also in the gastrointestinal tract (79).

In comparison to the abovementioned biomarkers, calprotectin has been more widely studied in CF. A study looking at children with CF found elevated fecal calprotectin levels and rectal nitric oxide production in majority of the subjects, indicating that intestinal inflammation is a major feature in CF (79). These values fell significantly after administration of the probiotic *Lactobacillus* GG, suggesting that the intestinal microbiota plays a role in CF intestinal inflammation (79). This was supported by Werlin and co-workers (78), who used wireless capsule endoscopy (WCE) and fecal calprotectin to investigate intestinal inflammation in children with CF. Fecal calprotectin was elevated only in pancreatic insufficient (PI) subjects, whereas WCE showed a high prevalence of small bowel injury in both PI and pancreatic sufficient (PS) children. The authors suggested that these findings support a “CF enteropathy” that is a primary feature of the CF phenotype and that its inflammatory component (as reflected by fecal calprotectin) changes with the degree of exocrine pancreatic impairment (78).

Nevertheless, it has been suggested that fecal sampling assessing intestinal inflammation in CF may potentially give false positive results due to the cross-reaction of ingested sputum proteins with intestinal inflammatory markers. However, this is likely a minor confounder to the significantly elevated fecal calprotectin detected in patients with CF (80).

In contrast, results from a recent study showed that unlike calprotectin, fecal S100A12 levels were not elevated in children with CF when compared to healthy controls (81).

## M2-Pyruvate Kinase

### Role in health

Pyruvate kinase (PK) is a key enzyme in the glycolytic pathway that catalyzes the conversion of phosphoenolpyruvate into pyruvate with eventual ATP production (82). It is expressed in all cells (83) and exists as dimeric and tetrameric isotypes in humans (84). The tetrameric (M1) type is found in skeletal muscles, heart, and brain (84), while the dimeric (M2) form is expressed by all rapidly dividing cells (both neoplastic and non-neoplastic) (83) (Table 2).

### Role in intestinal disease

Increased concentrations of fecal M2-PK are found in patients with CRC and M2-PK has been proposed as a potential screening tool for this cancer, with a sensitivity of 73% and specificity of 78% (85). Other studies have reported enhanced M2-PK activity in neutrophils in patients with polytrauma (86) and chronic cardiac failure (87). The role of M2-PK in gastrointestinal inflammation is unraveling (84), with several studies reporting its potential as a novel marker of intestinal inflammation.

#### Inflammatory bowel disease

In active IBD, there is increased intestinal epithelial cell turnover and rapid division (84). Hence, given the relationship of M2-PK to cell division, it has been postulated that fecal M2-PK concentrations are elevated in IBD patients (83). This has been supported by several studies.

Chung-Faye and co-workers (83) found fecal M2-PK to be significantly elevated in 81 adults with IBD and 7 with CRC when compared to 43 with IBS. Using a cutoff of 3.7 U/mL, fecal M2-PK had a sensitivity of 73% and specificity of 74% when used as a marker of organic gastrointestinal disease. Furthermore, M2-PK levels were greater in IBD patients with active compared to inactive disease. Their results also showed a high correlation between fecal M2-PK and calprotectin.

A pediatric study reported similar findings of significantly higher PK immunoreactivity in IBD patients (143.7 ± 24.6 U/g) when compared to healthy controls (1.2 ± 0.4 U/g; *P* < 0.00001) (82). Using the manufacturer recommended cutoff of 4 U/g, sensitivities of 94.3 and 100% were found for UC and CD, respectively. When a second cutoff of 5 U/g was used, false positives were reduced. However, specificity fell from 97.1% (cutoff of 4 U/g) to 94.3% (cutoff of 5 U/g) and sensitivity for CD fell to 94.1%. Regardless, the high sensitivity and specificity reflect the potential use of the fecal M2-PK test in pediatric IBD.

A more recent multicentre cohort study compared the ability of four fecal markers (calprotectin, lactoferrin, M2-PK, and S100A12) to predict the outcome in severe acute pediatric UC (88). Although all four markers reflected disease severity by their very elevated fecal values, only M2-PK had sufficient ability to predict corticosteroid treatment failure and the need for second-line therapy, presenting the potential for fecal M2-PK testing to be incorporated into clinical practice with further research. However, it was still inferior to the Pediatric UC activity index (PUCAI).

#### Pouchitis

Two studies looking at ileal pouch-anal anastomosis (IPAA) in patients with UC and familial adenomatous polyposis (FAP) who underwent restorative proctocolectomy found that those with pouchitis had significantly higher fecal M2-PK levels (89, 90). Johnson and colleagues (90) found that fecal M2-PK could differentiate between non-inflamed and inflamed pouches with a sensitivity and specificity of 80 and 70.6%, respectively. Moreover, fecal M2-PK levels correlated significantly with disease activity indices, endoscopic and histological appearances, as well as the degree of neutrophilic infiltration (90). These findings were mirrored in the earlier study despite a smaller sample size (89).

## Short-Chain Fatty Acids

Short-chain fatty acids are produced when colonic microflora ferment complex carbohydrates that are not absorbed in the small intestine (91) (Table 2). The main SCFAs liberated in the colon are propionate, acetate, and butyrate and their production can be altered by diet and rate of transit (3). The type of substrates derived from a person’s diet influences the production of SCFA. For instance, pectin is a particularly good source of acetate, while starch, oat, and wheat bran give rise to high amounts of butyrate (91). Antibiotics, especially those effective against Gram-negative and anaerobic bacteria, can also alter colonic SCFA production by reducing the fermentative capacity of the intestinal microflora (91).

### Role in health

Short-chain fatty acids have a wide range of actions. They are absorbed and metabolized rapidly by colonocytes, providing 60–70% of their energy requirements (91). They also regulate fluid and electrolyte uptake via activation of apical Na^+^/H^+^ exchange (91). Their presence in the colon lowers the pH, thus preventing the overgrowth of pH-sensitive pathogenic bacteria. Human rectal SCFA infusions have also shown to increase splanchnic blood flow and decrease gastric tone (3).

Butyrate, in particular, plays an important role in intestinal health. It has been shown to have a trophic effect on colorectal and ileal mucosal cells but despite this, is able to maintain normal colonic phenotype via growth arrest, differentiation, and apoptosis, thereby lowering the risk of malignancy. Importantly, butyrate enhances the gastrointestinal innate immunity by acting as a relay for transducing information from the luminal environment to the mucosal immune system via up-regulation of TLR expression (92). These TLRs enable the epithelium to differentiate commensal flora from pathogens, via recognition of bacterial molecular patterns called PAMPs and induce the transcription of a panel of genes mediating immune and inflammatory responses (92).

### Role in intestinal disease

#### Inflammatory bowel disease

Butyrate has anti-inflammatory effects that are mediated by the inhibition of NF-κB (i.e., inhibiting NF-κB nuclear translocation) in human colonic epithelial cells, therefore suppressing the gene transcription of pro-inflammatory cytokines. Histone deacetylase inhibition is the proposed mechanism behind this reduction in NF-κB translocation (93). The anti-inflammatory effects of butyrate have been demonstrated in UC, where butyrate enemas resulted in improved clinical disease activity and histological inflammation (94). The same anti-inflammatory effect was seen in CD, where butyrate reduced the expression of pro-inflammatory cytokines by intestinal biopsy specimens from CD patients (95).

#### Diversion colitis

Following the formation of an ileostomy, or after proximal colectomy for conditions such as IBD, where there is surgical diversion of the fecal stream (91), diversion colitis can develop due to the reduction in luminal butyrate levels (96). This results from the loss of butyrate as an energy source for colonocytes as well as a lack of its trophic effects on the colon (91). The anti-inflammatory effects of SCFAs were reiterated in a study which showed that SCFA-irrigation reversed the mucosal abnormalities in patients with diversion colitis (97).

#### Diarrheal disorders

Short-chain fatty acids have been found to have a role in diarrheal disorders, in accordance with their ability to stimulate fluid and electrolyte uptake. A study on cholera in children found that including rice starch in oral rehydration salts (ORS) resulted in faster clinical recovery (98). This was associated with striking increases in fecal bacterial concentrations and SCFA levels with time, indicating that this therapeutic effect might be mediated by SCFA enhancing sodium and water absorption and providing colonocytes with energy (98).

Short-chain fatty acids have also been implicated in the development of antibiotic-associated diarrhea (AAD). A study aimed at elucidating the pathogenesis of AAD found very low SCFA production in AAD patients versus controls (99). It is unclear whether AAD is secondary to impaired colonic fermentation resulting in decreased SCFA-stimulated sodium and water absorption, or the reduction in SCFA production is due to decreased colonic bacterial count (99).

#### Cystic fibrosis

It has been hypothesized that malabsorption of carbohydrates and to a lesser extent, protein, that occurs in CF associated with pancreatic insufficiency, can serve as substrates for fermentation by colonic microflora, thus leading to an increase in SCFA production and excretion in stool (100). Increased fecal SCFA output (50 ± 30 mmol/day) was found in patients with pancreatic insufficiency secondary to chronic pancreatitis unrelated to CF when compared to healthy individuals (10–20 mmol/day) (101). This finding of increased SCFA production in a maldigestive state similar to CF might indicate mechanisms other than pancreatic insufficiency in determining fecal SCFA output in CF patients. Notably, SCFA derivatives have been shown to correct the ΔF508-CFTR mutation *in vitro*, by correcting the inability of the ΔF508-CFTR protein to traffic to the cell surface membrane and by activating alternate chloride transport pathways (102).

## Practical Aspects of Fecal Biomarkers

Of the markers discussed, all except SCFA can be measured in stool via sandwich ELISAs, which are mostly commercially available and therefore easily performed in a routine laboratory.

There is limited information on the stability of defensins in stool. In preparation for one study (19), the authors looked at three sample storage conditions – 48 h at room temperature, 1 week at 4°C, and 3 months at −80°C – and observed no significant difference in HBD2 levels in stool. These results suggest that fecal samples can be stored in a patient’s home refrigerator for up to a week prior to laboratory testing, therefore enhancing the clinical utility of the test. On the contrary, no information regarding the stability of cathelicidin in stool has been found.

Lactoferrin, calprotectin, and S100A12 are stable in stool at room temperature for up to 4, 7, and more than 7 days, respectively (58). In addition to the resistance of lactoferrin to proteolysis in stool, it is unaffected by multiple freeze-thaw cycles (84). Calprotectin and S100A12 also have a homogenous distribution in stool (58). These features increase the convenience and acceptability of sample collection to children and parents, with the potential to use regular mail for the S100 proteins. They also ensure the accuracy of measurement via ELISA in the laboratory, further enhancing their desirability as fecal biomarkers.

M2-pyruvate kinase is stable for 2 days at room temperature in stool (84). Commercial ELISAs have been previously developed and validated for use in CRC screening and are thus readily available.

Osteoprotegerin, on the other hand, decays rapidly in stool within 24 h at room temperature (54). This means that samples have to be freshly collected and kept frozen at −80°C until tested.

Measurement of SCFA in stool is done via high-performance liquid chromatography (HPLC), therefore requiring additional expertise and laboratory equipment than an ELISA. SCFA, both volatile and non-volatile, are found to be stable in stool for at least 7 days at room temperature if samples are treated with 70% ethanol (103). Fecal samples are suggested to be treated immediately after defecation (103), which may be inconvenient for patients. A further drawback of measuring fecal levels of SCFA is that <5% of SCFA produced in the colon is excreted in stool (91). Moreover, fecal SCFA can be altered by diet and rate of transit, and therefore may only be useful in reflecting changes in excretion but not in production. Alternative methods for measuring SCFA are the breath gas test and peripheral venous SCFA. However, these methods are general indicators of SCFA fermentation and not intestinal specific (3). These disadvantages presented in the literature affect the feasibility and clinical utility of measuring SCFA in stool to a fair extent.

## Areas for Consideration and Future Research

Currently, the clinical role of fecal biomarkers is better established in diseases like IBD, with a paucity of information in other states of gastrointestinal disease and inflammation. More research into these biomarkers has to be performed before they can be used to accurately define the basic biological processes and pathogenesis of disease. With more research to further our understanding of these biomarkers, there is the potential for them to be incorporated into clinical practice. Although endoscopy with tissue biopsies is at present the only accurate means of detecting intestinal inflammation (58), testing of fecal biomarkers can be used as an initial screening test to determine the need for more invasive investigations. Their non-invasive nature is also especially valuable in the pediatric population. Furthermore, the analysis of these biomarkers directly from stool provide intestinal-specific information as opposed to the commonly used serum markers such as C-reactive protein (CRP) and ESR, which reflect a summation of systemic inflammatory responses (58). Therefore, fecal biomarkers may be a viable option for monitoring disease activity in the follow-up of patients and assessing their response to anti-inflammatory therapies. The current lack of age-related reference ranges of these biomarkers in healthy children, apart from calprotectin and S100A12, needs to be addressed. In addition, with future work to more clearly define healthy from disease levels, these biomarkers can be used for risk stratification in patients to direct therapy and predict clinical outcomes.

In addition to protein biomarkers, intestinal microbes and microbial products also have the potential to be used as disease markers. However at present, more research into their role in healthy and disease states has to be done. Therefore, they are less applicable to clinical practice in the immediate future as compared to protein biomarkers. With reducing costs and the automation of high-throughput sequencing, microbes and their products show great promise as disease markers for future clinical use (104). A further advantage is that determining specific compositions of gut microbiota in disease states may provide more insight into the causes of such diseases. This is in contrast to quantifying protein biomarkers, which reflect more on the effects of disease states.

## Conclusion

There are various biomarkers, a few of which have been reviewed above, that may be useful in providing insight into the role of intestinal health and disease, and the development of non-gastrointestinal conditions associated with intestinal dysbiosis. There is a current lack of literature on the normal ranges as well as age-related changes of these biomarkers in healthy children, which limit the applicability of these fecal biomarkers in a general clinical setting.

## Conflict of Interest Statement

The authors declare that the research was conducted in the absence of any commercial or financial relationships that could be construed as a potential conflict of interest.
